# Cervical Spine Manipulation and Causation of Cervical Artery Dissection: A Review of 10 Case Reports

**DOI:** 10.7759/cureus.62845

**Published:** 2024-06-21

**Authors:** Steven P Brown

**Affiliations:** 1 Integrative/Complementary Medicine, Brown Chiropractic & Acupuncture, PC, Gilbert, USA

**Keywords:** cerebrovascular stroke, medicolegal analysis, causation, internal carotid artery dissection, vertebral artery dissection, case report, stroke, chiropractic, cervical spine manipulation, cervical artery dissection

## Abstract

Recent media coverage of high-profile cases of cervical artery dissection (CAD) has ignited the discussion about the role of cervical spine manipulation (CSM) in causing cervical artery dissection.

However, research does not support a causal association between cervical spine manipulation and cervical artery dissection in a healthy cervical spine. The objective of this study was to review the 10 most recent case reports of cervical spine manipulation and cervical artery dissection for convincing evidence of the causation of cervical artery dissection by cervical spine manipulation.

Nine of 10 case reports showed no convincing evidence of a causal relationship between cervical spine manipulation and cervical artery dissection. The 10th case report was exceptional as the CSM was contraindicated by pre-existing cervical spine pathology.

We conclude that these 10 case reports provide no convincing evidence of the causation of cervical artery dissection by cervical spine manipulation in a healthy cervical spine. One case report demonstrated that cervical spine manipulation can cause cervical artery dissection when performed in the presence of pre-existing cervical spine pathology. Therefore, we conclude that practitioners should exclude cervical spine pathology before performing cervical spine manipulation.

## Introduction and background

Cervical arteries include the vertebral artery (VA) and the internal carotid artery (ICA). Cervical artery dissection (CAD) may refer to vertebral artery dissection (VAD), internal carotid artery dissection (ICAD), or both.

Cervical artery dissection

**Figure 1:**
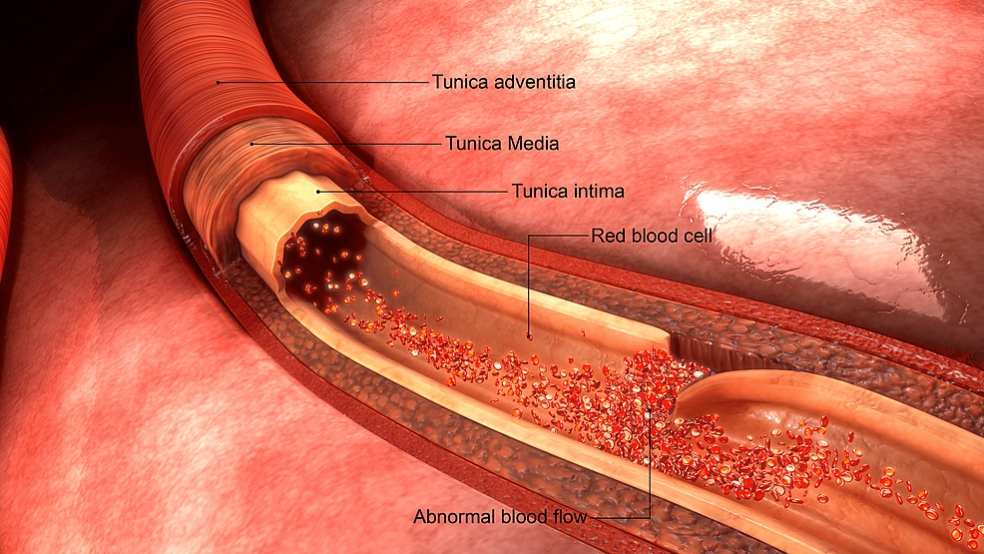
Anatomy of the arterial wall with intimal dissection Image credit: Shutterstock

The arterial wall consists of three layers: the tunica intima (innermost layer), the tunica media (middle layer), and the tunica adventitia (outermost layer). VAD and ICAD are reported to arise from a flap-like tear or "dissection" of the tunica intima. The dissected arterial wall causes abnormal blood flow and heals by forming a thrombus (Figure 1).

The vertebral artery has four segments: V1-V4. VAD can be either extracranial (V1, V2, or V3) or intracranial (V4). Extracranial dissections that follow CSM are considered to usually involve the distal extracranial segment near C1 and C2 (V3). The internal carotid artery has seven segments, C1-C7, only one of which is extracranial (C1). ICADs following CSM are considered to usually involve the extracranial segment, which is also near C1 and C2 just below the skull. The most characteristic symptoms of CAD are neck pain and headache [1].

Cervical artery dissection and cervical spine manipulation

Research does not support a causal association between cervical spine manipulation (CSM) and CAD. CSM causes little or no strain on the cervical arteries [2,3]. In a statement from the American Heart Association and American Stroke Association, Biller et al. [4] found that biomechanical evidence is insufficient to establish the claim that CSM causes CAD and recommended that practitioners should strongly consider CAD as a presenting symptom prior to CSM. Church et al. [5], a group of neurosurgeons from Penn State Hershey Medical Center, found no convincing evidence that CSM can cause CAD in an otherwise healthy artery.

While it is not plausible that CSM could cause CAD in a healthy cervical artery, it is plausible that CSM could cause CAD in a susceptible cervical artery [6]. A susceptible cervical artery would be one predisposed to dissection by environmental or inherited risk factors. Inherited risk factors include connective tissue disorders such as vascular Ehlers-Danlos syndrome [7].

Cervical artery dissection and stroke

The onset of symptoms of ischemic stroke immediately after CSM is often assumed to be the onset of CAD [8]. However, research shows that in cases of stroke immediately following CSM, the patient likely had an existing CAD before the CSM [9,10]. Biller et al. [4] recommended that practitioners should strongly consider CAD as a presenting symptom prior to CSM.

Even if CSM did cause CAD in a susceptible artery, it would not be likely to cause immediate stroke by a thromboembolic or thrombotic mechanism. It is not plausible for a thrombus to instantly form, dislodge, travel to the brain, and cause a stroke within seconds or minutes of CSM [6]. The normal clotting time is 4-10 minutes [11]. It would then take more time for a thrombus to accumulate, dislodge, and embolize to occlude a smaller artery that supplies the brain, resulting in a thromboembolic stroke. Moreover, the formation of large thrombi that can occlude an artery, resulting in a thrombotic stroke, also requires time.

The immediate consequence of CAD from CSM would be sudden neck pain and/or headache, a brief syncope, and perhaps nausea, vertigo, and tinnitus [12]. A stroke is not likely to occur immediately, and if it occurs at all, it would not be until hours or days later due to the enlargement of the dissection or propagation of a thrombus.

Objective

Recent cases involving CSM and CAD have received high-profile media coverage. Cases in California in 2016 [13] and Georgia in 2022 [14] have ignited the discussion about the role of CSM in causing CAD. However, research does not support a causal association between CSM and CAD. The objective of this study was to review the most recent 10 case reports of CSM and CAD for convincing evidence of the causation of CAD by CSM.

As CAD can have serious consequences, establishing the causation of CAD by CSM with randomized controlled trials is infeasible [15]. As CAD is rare, establishing the causation of CAD by CSM with epidemiological studies is difficult. Therefore, the standard for the establishment of causation used in this study is the medicolegal standard of more likely than not [16].

## Review

Methodology

In May 2023, a search of the PubMed database from January 2021 to April 2023 was conducted to identify the 10 most recent English-language case reports or conference abstracts using the key terms "chiropractic," "cervical spine manipulation," "case report," and "cervical artery dissection." Studies were included if an adverse event following CSM was described as a CAD. Case reports were reviewed to determine if the adverse event following CSM was a CAD and to determine if there was any convincing evidence that CSM caused CAD. Retrospective commentaries or letters to the editor concerning the case reports were excluded.

Results

The literature search found nine studies containing 10 case reports (Table 1).

**Table 1:** Summary of cases selected

Case #	Year	Author	Study type	Country
1	2023	Yeung et al. [17]	Conference abstract	USA
2	2022	Chen et al. [18] (case 1)	Case series	China
3	2022	Chen et al. [18] (case 2)	Case series	China
4	2022	Arning et al. [19]	Case report	Germany
5	2022	Abidoye et al. [20]	Conference abstract	USA
6	2021	Yap et al. [21]	Case report	China
7	2021	Xia et al. [22]	Conference abstract	USA
8	2021	Lindsay et al. [23]	Case report	USA
9	2021	Monari et al. [24]	Case report	Italy
10	2021	Ramos et al. [25]	Case report	Brazil

Review

Significant case report information is summarized in Table 2.

**Table 2:** Summary of significant case report information CSM = cervical spine manipulation, CAD = cervical artery dissection, VA = vertebral artery, ICA = internal carotid artery, V1, V2, V3 = vertebral artery segments 1, 2, 3, C1, C3 = internal carotid artery segments 1, 3

Case #	Age/sex	Onset of symptoms of CAD	Area of CAD
1	48/female	Neck pain before CSM	Right V1
2	51/male	Right neck pain 2 days after CSM	Right C3
3	55/male	Right neck pain 19 hours after CSM	Right C3
4	47/female	Right neck pain 2 weeks before CSM	Right V2
5	40/male	Neck pain and migraines 2 months before CSM	Right C1
6	35/male	Neck pain 2 weeks before CSM	Left ICA
7	44/male	Neck pain before CSM	Left V2, right V3
8	47/male	Neck pain 6 years before CSM	Left V3
9	39/female	Tension headache/migraines before CSM	Right V2
10	48/female	Neck pain before CSM	Bilateral VA

Case 1: Yeung et al. (2023) [17]

Yeung et al. [17] reported that a "48-year-old female went to a chiropractor for chronic neck pain and developed right-sided weakness, nausea, dizziness, and vomiting immediately after neck manipulation." Imaging showed occlusion of the V1 segment of the right vertebral artery and cerebellar stroke.

The adverse event immediately following CSM was the cerebellar stroke, not the CAD. Right-sided weakness, nausea, dizziness, and vomiting are symptoms of cerebellar ischemia, not right VAD. The neck pain prior to the CSM is consistent with a CAD being present prior to CSM, not caused by CSM.

Even if CSM had caused the CAD, it is not biologically possible for a thrombus large enough to occlude the vertebral artery to form immediately [6]. Therefore, the CAD was likely pre-existing to CSM. While an existing thrombus may have been aggravated by the CSM, it was not caused by the CSM. In this case, it is plausible that CSM may have suddenly repositioned an already large thrombus in such a way that it blocked the V1 segment of the right vertebral artery, resulting in thrombotic ischemic stroke from vascular occlusion [26]. The practitioner failed to exclude CAD and performed CSM when it was contraindicated [7]. So, while thrombotic stroke may have been causally related to the CSM, the CAD was not.

Cases 2 and 3: Chen et al. (2022) [18]

Chen et al. [18] reported that "a 51-year-old man with a history of mild hypertension noted new-onset right neck pain two days following chiropractic manipulation." Imaging revealed dissection of the C3 segment of the right ICA and right-sided stroke.

Chen et al. [18] also reported a second case in which "a 55-year-old man with a history of cigarette smoking, no other cerebrovascular risk factors, received chiropractic cervical manipulation 1 day prior to presentation to the emergency department with new onset of left hemiparesis, facial paralysis, right neck pain, and dysarthria lasting for 5 hours." Imaging revealed dissection of the C3 segment of the right ICA and right-sided cerebral stroke.

In these two case reports, the symptoms that prompted the patients to seek CSM were not documented. In the first case, neck pain started two days after CSM. In the second case, neck pain started 19 hours after CSM.

In these two cases, there was no adverse event immediately following CSM. As there was no neck pain, headache, or ischemic symptoms noted immediately after CSM, it is not likely that CSM caused the ICA dissection or the stroke. Furthermore, the C3 segment of the ICA is intracranial and has not been identified as an area for strain by CSM.

Case 4: Arning et al. (2022) [19]

Arning et al. [19] reported the case of a 47-year-old female with a two-week history of non-traumatic right neck pain who had increased, severe right neck pain immediately after CSM, and paresis of the right deltoid muscle and hypalgesia in the right C3 and right C4 dermatomes. MRI revealed a dissection of the V2 segment of the right vertebral artery.

The adverse event immediately following CSM was a stroke, not a CAD. Paresis and hypalgesia are symptoms of brain ischemia, not right VAD. The right neck pain prior to the CSM is consistent with a right VAD being present prior to CSM, not caused by CSM.

Prior to CSM, cervical spine disc herniation had been ruled out by MRI. Upon review, the pre-CSM MRI also showed dissection of the right V2 segment, which had initially been overlooked by the radiologist. The practitioner performed CSM when it was contraindicated. Therefore, while the CSM may have caused the ischemic stroke by a thromboembolic mechanism, the CSM did not cause the CAD.

Case 5: Abidoye et al. (2022) [20]

Abidoye et al. [20] reported, "This is a 40-year-old male with a medical history of migraine headaches and cervicalgia, evaluated for a sudden onset of headache, associated with nausea, vomiting, blurred vision, and dizziness, two months after a chiropractic manipulation. He also reported rigorous exercise and sexual intercourse prior to the headache onset. Vital sign is significant for a 10/10, non-radiating right-sided headache. Neurological examination revealed right ptosis and miosis. Labs were unremarkable. CTA of neck showed tapering of the right ICA with near occlusion at the skull base." No imaging evidence or diagnosis of stroke was documented. However, with ischemic symptoms of nausea, vomiting, blurred vision, dizziness, right ptosis, and right miosis, it is likely that this patient suffered a stroke.

In this case, there was no adverse event immediately following CSM, and the most recent CSM was two months prior to the onset of symptoms. As there was no neck pain, headache, or ischemic symptoms noted immediately after CSM, it is not likely that CSM caused the ICA dissection or the stroke.

The patient's medical history of neck pain and headaches are risk factors for CAD. If there was existing right ICA dissection, it is plausible that rigorous exercise and sexual intercourse could have dislodged a loosely adherent ICA thrombus and caused immediate stroke by a thromboembolic mechanism. However, this is not possible to determine as the temporality from exercise and intercourse to ischemic symptoms of stroke was vaguely documented as "prior to."

Case 6: Yap et al. (2021) [21]

Yap et al. [21] reported a 35-year-old male who presented with a two-day history of expressive dysphasia and a one-day history of right-sided weakness. The patient reported having CSM for pain relief sometime in the prior two weeks. Imaging showed left ICA dissection and left middle cerebral artery stroke. The dissected segment of the left ICA was not documented.

In this case, there was no adverse event immediately following CSM. As there was no neck pain, headache, or ischemic symptoms noted immediately after CSM, it is not likely that CSM caused the ICA dissection or the stroke.

Case 7: Xia et al. (2021) [22]

Xia et al. [22] reported a case of a 44-year-old male with chronic neck pain who reported sudden-onset left homonymous hemianopia after CSM a few days prior. The patient reported progression from a left homonymous hemianopia to a left homonymous inferior quadrantanopia. Imaging revealed bilateral VAD at the left V2 and right V3 segments, and right medial occipital lobe stroke. The authors noted that a right posterior communicating artery stroke was likely embolic from the right V3 and left V2 dissections. They also noted that the patient likely had a migrating embolus as evidenced by the progression from a homonymous hemianopia to a quadrantanopia.

The adverse event immediately following CSM was the stroke, not the CAD. Left homonymous hemianopia is a symptom of brain ischemia, not VAD. The neck pain prior to the CSM is consistent with VAD being present prior to CSM, not caused by CSM.

Even if CSM had caused the CAD, it is not biologically possible for a thrombus to instantly form and dislodge to cause sudden-onset thromboembolic stroke [6]. Therefore, the CAD was likely pre-existing to CSM. While an existing thrombus may have been aggravated by the CSM, it was not caused by the CSM. In this case, it is possible that CSM dislodged a loosely adherent vertebral artery thrombus to cause thromboembolic stroke [26]. The practitioner failed to exclude CAD and performed CSM when it was contraindicated [7]. So, while thromboembolic stroke may have been causally related to the CSM, the CAD was not.

Case 8: Lindsay et al. (2021) [23]

Lindsay et al. [23] reported a case of a 47-year-old male who presented with left neck pain and headache. His medical history was notable for dyslipidemia and a cerebellar stroke six years prior. Imaging revealed dissections of the left vertebral artery extending from the origin of the artery to the V3 segment. The patient also had a dissection of his right renal artery. There was no evidence of a stroke.

Six years prior, the patient had presented with a one-week history of left neck pain and headache, as well as left facial numbness and dizziness. The pain was not relieved with ibuprofen and previously been evaluated and treated by a chiropractor. Imaging done six years prior showed no evidence of CAD but did show a left cerebellar stroke.

There is no plausible biological mechanism by which CSM six years prior could cause a current VAD. Therefore, it is not likely that there was a causal relationship between CSM and CAD in this case.

Ultimately, the patient was diagnosed with vascular Ehlers-Danlos syndrome, a disorder that causes connective tissue weakness and makes a patient susceptible to arterial dissection. This diagnosis is consistent with the left VAD and right renal artery dissection.

Case 9: Monari et al. (2021) [24]

Monari et al. [24] reported a case of a 39-year-old pregnant female with a history of tension headaches presenting with vertigo, vomiting, nystagmus, dizziness, and hindrance in the execution of fine movements of the right arm. The patient reported having CSM by an osteopathic specialist "in the days preceding the beginning of the symptoms." Imaging showed a dissection of the V2 segment of the right vertebral artery and a right-sided stroke.

In this case, there was no adverse event immediately following CSM. As there was no neck pain, headache, or ischemic symptoms noted immediately after CSM, it is not likely that CSM caused the right vertebral artery dissection or the stroke. Medical history of headache prior to the CSM is consistent with a VAD being present prior to CSM, not caused by CSM. Pregnancy is also a risk factor for CAD.

Case 10: Ramos et al. (2021) [25]

Ramos et al. [25] reported a case of a 48-year-old female with a history of chronic neck pain who experienced sudden neck pain and generalized weakness during CSM. Imaging showed bilateral VAD and occlusion and bilateral acute cerebellar stroke. There was also tetraplegia noted at the C5 sensory level, C5 and C6 vertebral fracture, spinal cord injury, epidural hematoma, and acute disc herniation.

There is convincing evidence that CSM caused CAD and stroke in this case. This case is exceptional as the CSM was contraindicated by pre-existing cervical spine pathology. Cervical spine bony ankylosis was noted which existed prior to the CSM. The CSM appears to have been a posterior-anterior manipulation of the cervical spine at the level of C5-C6, which was contraindicated due to the presence of the bony ankylosis [27].

The practitioner failed to exclude cervical spine pathology and performed CSM when it was contraindicated. The spinal pathology in this case could have been diagnosed with a cervical spine X-ray examination.

As the Ramos et al. [25] study provided limited case information, a case report from Macêdo et al. [28] provides additional information on this exceptional case.

"A 47-year-old Afro-Brazilian woman with long-standing back pain sought chiropractic care for symptomatic relief. Until then, she had never consulted a doctor to treat her axial pain and was not aware of having any specific spinal pathology. Since childhood, she had a moderate cognitive deficit, which probably compromised her ability to adequately describe the pain and, thus, led the family to seek medical advice. During her last session of spinal manipulation, she mentioned new-onset paresthesia beginning on the upper limbs and progressing to the lower limbs. Her complaint was disregarded, and the session continued, at the end of which she was unable to stand. Urinary retention ensued a little after. The patient was referred to our service only a week after, completely bedridden. Spine MRI revealed a transdiscal fracture at C5-C6, resulting in critical stenosis and compressive myelopathy. CT angiography revealed traumatic thrombosis of the vertebral arteries emerging on this level. Whole spine-imaging evidenced multiple syndesmophytes giving a characteristic bamboo spine appearance, as well as ankylosis in sacroiliac joints, uncovering the diagnosis of ankylosing spondylitis. She underwent laminectomy from C2 to C6 and arthrodesis from C2 to T2 for spine stabilization but did not recover mobility. Even though a systematic review did not find an increased risk of significant adverse events related to spine manipulation therapy, there have been descriptions of vertebral fracture following a session on patients with ankylosing spondylitis and unsuspected multiple myeloma."

Medicolegal causation analysis

Medicolegal causation can be established as more likely than not if plausibility, temporality, and lack of a more probable alternative explanation are present [16]. A medicolegal analysis of these 10 cases follows.

Plausibility

In cases 1-9, it is not plausible that CSM could have caused the CAD, as there is no convincing evidence that CSM can cause CAD in a healthy cervical spine [5]. In case 10, it is plausible due to the presence of pre-existing cervical spine pathology.

Temporality

In cases 1-9, there was not a close temporal association (immediate) between CSM and the onset of the symptoms of CAD (neck pain and/or headache). In case 10, although neck pain was present before the CSM, the unique nature of this sudden catastrophic injury makes a close temporal association of CSM and CAD more likely than not.

Lack of a More Probable Alternative Explanation

In cases 1-9, there is a more probable alternative explanation of the cause of the CAD. Spontaneous CAD unrelated to CSM is a more probable explanation. In case 10, the unique nature of this sudden catastrophic injury makes an alternative explanation unlikely.

Table 3 presents a summary of our medicolegal causation analysis.

**Table 3:** Summary of medicolegal causation analysis CSM = cervical spine manipulation, CAD = cervical artery dissection

Case #	Plausibility of CAD from CSM	Immediate temporality of CSM and symptoms of CAD	Lack of more alternative explanation	Causation more likely than not
1	No	No	No	No
2	No	No	No	No
3	No	No	No	No
4	No	No	No	No
5	No	No	No	No
6	No	No	No	No
7	No	No	No	No
8	No	No	No	No
9	No	No	No	No
10	Yes	Yes	Yes	Yes

Limitations

Three of the studies reviewed were conference abstracts, and a full study was not available. It is possible that not all case information was included in the conference abstract.

Another limitation is that only one literature database was searched. Future research could be improved by searching databases from physiotherapy, osteopathic, naturopathic, neurology, and emergency medicine professions. Other databases that could also be searched include EBSCOhost, Scopus, Web of Science, and Google Scholar.

## Conclusions

We conclude that nine out of the 10 case reports of CSM and CAD did not provide convincing evidence of the causal relationship between CSM and CAD. Only one case report provided convincing evidence of a causal relationship between CAD and CSM. This case was exceptional as the CSM was contraindicated by pre-existing cervical spine pathology. Therefore, we conclude that practitioners of CSM should exclude cervical spine pathology before performing CSM.
